# Z-score model of foetal ascending aorta diameter distensibility

**DOI:** 10.3389/fcvm.2022.858235

**Published:** 2022-08-11

**Authors:** Fuli Chen, Shi Zeng, Aijiao Yi, Lihua Chen, Dan Zhou, Yushan Liu, Longmei Yao

**Affiliations:** ^1^Department of Ultrasound Diagnosis, The Second Xiangya Hospital, Central South University, Changsha, China; ^2^Department of Ultrasound Diagnosis, The Central Hospital of Yueyang City, Yueyang, China

**Keywords:** fetal, aortic elastic properties, prenatal, aortic diameter distensibility, Z-score

## Abstract

**Objective:**

The purpose of this study is to establish Z-scores models of normal fetal ascending aorta diameter and diameter distensibility.

**Methods:**

The maximum systolic diameter (Dmax), minimum diastolic diameter (Dmin), and diameter distensibility of the sinotubular junction were measured and taken as dependent variables in 490 normal fetuses at 18–40 gestational weeks, and gestational age (GA), biparietal diameter (BPD), and femoral length (FL) were taken as independent variables. The data were subjected to regression analysis, and the best-fitting equations for the dependent variables based on the independent variables were determined. The fitting equations were then applied to construct the Z-scores models.

**Results:**

The Dmax, Dmin and Diameter Distensibility in normal fetuses between 18 and 40 weeks of GA could be evaluated by utilizing the Z-scores models. Dmax and Dmin increased significantly with increasing GA, BPD, and FL. Diameter distensibility, assessed as (Dmax–Dmin)/Dmin, decreased significantly with increasing GA, BPD, and FL.

**Conclusion:**

The Z-scores are valuable, and can be utilized as a potent supplement to the conventional approach as they can indirectly reflect the development of fetal ascending aortic elastic property.

## Introduction

In recent years, some studies have found that congenital heart disease and intrauterine growth retardation can induce aberrant arterial elasticity after birth and that this alteration have already existed during the fetal period (1–4). Abnormalities in arterial elastic function will have a profound impact on the development of the cardiovascular system over the course of an individual’s life. Therefore, prenatal assessment of arterial elastic function is essential for the monitoring of potentially impaired vascular function throughout pregnancy as well as early intervention and prevention after birth (5). Ultrasound has become the most effective means of prenatal examination due to its advantages of being non-invasive, economical and convenient. Studies attempted to objectively evaluate the elastic properties of fetal arteries through various methods and discovered that aortic elastic properties varied dynamically with increasing gestational age (GA) (6–8). There are many indices for evaluating the elastic properties of arteries *via* ultrasound, among which compliance, distensibility, the stiffness index, Peterson’s elastic modulus, and pulse wave velocity are commonly used (9, 10). Although the values of these indices are associated with blood pressure, there is no parameter regarding blood pressure in human fetuses due to the inaccessibility of fetal vessels, except when an invasive approach is adopted (11). In previous research on fetal arterial elastic properties, some scholars have explored differences in aortic inner diameter alterations to indirectly evaluate arterial elasticity (12, 13). However, there are only a few studies on this aspect at present, and these studies are limited by the sample size and different GAs of fetuses. As a result, we set out to evaluate the development trend of fetal arterial elastic characteristics by measuring the maximum systolic diameter (Dmax), the minimum diastolic diameter (Dmin) and the diameter distensibility of the aortic sinotubular junction, as well as to establish reference ranges for Dmax, Dmin and diameter distensibility in the hope that these could serve as simple tools for analyzing vessel function.

## Materials and methods

### Study population

A prospective observational study was conducted at the Central Hospital of Yueyang and the Second Xiangya Hospital of Central South University between May 1, 2020, and December 31, 2020. Healthy pregnant women attending an antenatal care clinic were enrolled in the study. The inclusion criteria were as follows: (1) singleton pregnancies; (2) specific GA based on sonographic dating in the first-trimester pregnancy; and (3) GA between 18 and 40 weeks. The exclusion criteria were as follows: (1) fetal structural or chromosomal abnormalities; (2) fetal growth restriction (fetuses with an estimated fetal weight (EFW) < the 10th percentile) or macrosomia (fetuses with an estimated EFW > the 90th percentile); and (3) poor image quality. This study was approved by the institutional review board for ethics at the Second Xiangya Hospital and The Central Hospital of Yueyang. All parents provided written consent.

### Data collected

Routine obstetrical ultrasound examination was performed by one investigator (Z. X) using GE Voluson E8 (GE Healthcare Ultrasound, Milwaukee, WI, United States) and E10 (GE Healthcare Ultrasound, Milwaukee, WI, United States) with probe RAB4-8-D (E8) and RM6C (E10). The participants underwent a standard sonographic examination for fetal anomaly screening and biometric measurements with the prenatal ultrasound examination specifications system ultrasound inspection (14). Following the measurement of biometric parameters, including biparietal diameter (BPD), head circumference (HC), femur length (FL), and abdominal circumference (AC). Multiple standard views of each fetal heart were obtained to evaluate the cardiac anatomy.

**FIGURE 1:**
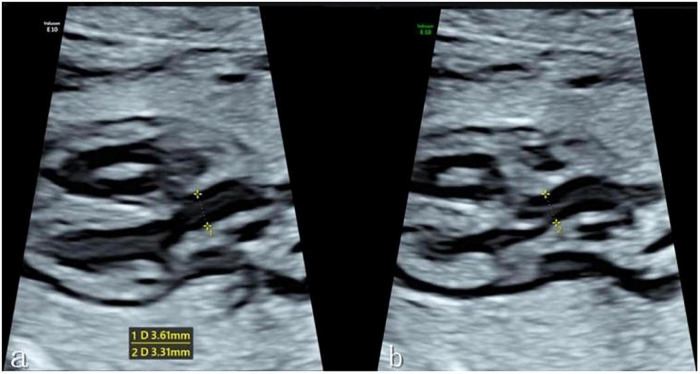
**(a)** Measurement of the Dmax of the ascending aortic sinotubular junction at the end of systole in the left ventricular outflow tract view. **(b)** Measurement of the Dmin of the ascending aortic sinotubular junction at the end of diastole. *Caliper.

The fetal left ventricular outflow tract view was then clearly displayed in the instrument fetal heart mode, with the ultrasound beam and ascending aorta long axis kept as vertical as possible to ensure a clear display of the echo of the tube wall. When the fetus had no obvious movement, one investigator (C.FL.) obtained a clear image of the left ventricular outflow tract section, and the Dmax and Dmin of the ascending aorta were evaluated at the sinotubular junction at the end of systole and end of diastole, respectively. End-systole is defined as the frame before the aortic valve closes, while end-diastole is defined as the frame before the aortic valve opens. When measuring the inner diameter of the sinotubular junction, the target was expanded to fill more than 1/3 of the screen, a caliper was placed on the tube wall, and the distance between inner edges was measured (see Figure 1). Each data point was measured three times before an average was determined. The ascending aorta diameter expansion rate was then computed as follows: Diameter Distensibility (100%) = (Dmax-Dmin)/Dmin*100%.

### Statistical analysis

The statistical analysis software SPSS 22.0 was employed. According to Devore’s statistical methods (15) and our team’s prior studies (16), the calculation of the Z-score can be condensed into three steps. Step 1 was to run the regression model and acquire the equation of the mean. Dmax, Dmin, and Diameter Distensibility were taken as dependent variables, while GA, BPD, and FL were taken as independent variables. Regression analysis was performed on the two, and the best-fitting regression equation was selected. Step 2 was to derive the fitted regression equation of the standard deviation (*SD*). In this step, GA, FL, and BPD were also the independent variables, while the *SD* of each dimension was the dependent variable. Finally, the Z-score was calculated as follows: (observed value-predicted value in step 1)/predicted *SD* in step 2. The P-P plots method was used to test the normality of the residuals and Z-scores. The intraclass correlation coefficient (ICC) was employed to assess the consistency between observers. Twenty cases were randomly selected among the subjects, 2 distinct ultrasonography examiners repeated the measurement at 30-min intervals to assess interobserver variability, and 20 samples were randomly selected at 30-min intervals to allow the same examiner to repeat the measurement to assess intraobserver variability.

## Results

**FIGURE 2:**
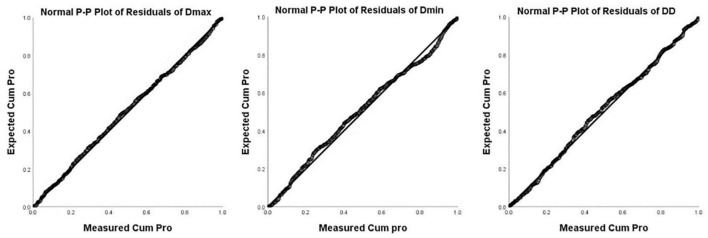
P-P Plot of residuals of Dmax, Dmin, and DD (Diameter Distensibility) based on gestational age.

**FIGURE 3:**
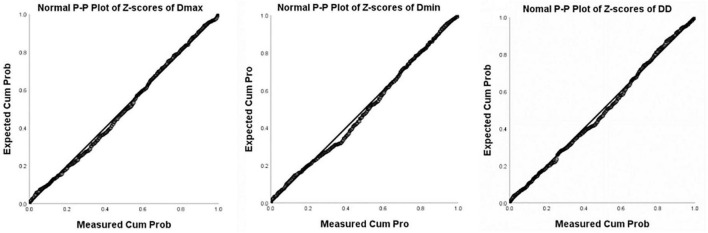
P-P Plot of Z-scores of Dmax, Dmin, and DD (Diameter Distensibility) based on gestational age.

**FIGURE 4:**
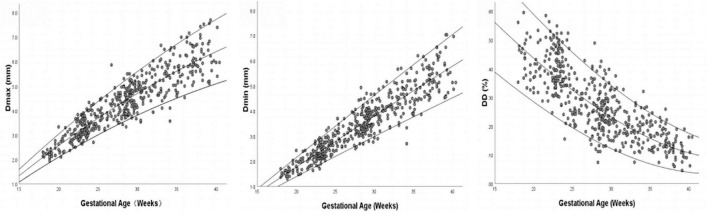
Scatter plot of Dmax, Dmin, and DD (Diameter Distensibility) based on gestational age (*n* = 490), with the 5th, 50th, and 95th percentiles superimposed.

**FIGURE 5:**
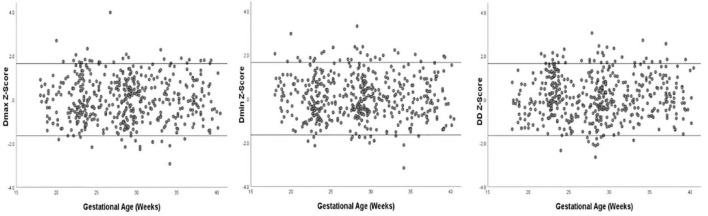
Scatter plot of Z-score distribution of Dmax, Dmin, and DD (Diameter Distensibility) based on gestational age (*n* = 490), with tram lines at 1.96 and −1.96.

Images of 504 fetuses were successfully acquired with satisfactory quality and were available for offline analysis. However, 14 data sets were later excluded since the aortic valve was not visible. Therefore, the remaining 490 data sets with successful measurements were used for statistical analysis. The majority of the participants (270 [55.10%]) were nulliparous. The mean maternal age ± *SD* was 26.38 ± 6.77 years (range, 18–43 years). All were healthy Chinese women residing in Hunan, China. Intraobserver variability and interobserver variability in the observation index measurements were both found to have good agreements, as shown in Table 1. The Dmax and the Dmin increased significantly as GA increased, while diameter distensibility decreased (*P* < 0.001). The best-fitting equations for predicted values and SDs based on independent variables (GA, BPD, and FL) are shown in Tables 2, 3. These equations were validated by constructing a Z-score for each variable. The normality of the residuals and Z-scores were demonstrated in a P-P plot (see Figures 2, 3). In addition, the Z-scores were equally distributed above and below 0 across the entire range of GAs, as well as following a standard normal distribution. The number of Z-scores distributed outside the range of ± 2 *SD* was as expected: approximately 10% of the values, as seen in Figures 4, 5.

**TABLE 1:** The results of the repeatability test.

	Parameter	ICC
Intraobserver consistency	Dmax	0.970
	Dmin	0.930
	Diameter distensibility	0.976
Interobserver consistency	Dmax	0.991
	Dmin	0.991
	Diameter distensibility	0.931

ICC, intraclass correlation coefficient; Dmax, maximum systolic diameter; Dmin, minimum diastolic diameter.

**TABLE 2:** Regression models for the prediction of the mean of Dmax, Dmin, and diameter distensibility based on GA, BPD, and FL.

Parameter	Model derived from regression analysis	*R*	*P*
**GA (weeks)**			
Dmax (mm)	Y = −2.99 + 0.32*GA-2.14*GA^2^*10^–3^	0.905	<0.001
Dmin (mm)	Y = −2.41*GA-0.210*GA-3.39*GA^3^*10^–6^	0.92	<0.001
Diameter distensibility (100%)	Y = 0.98–0.03*GA + 4.48*GA^3^*10^–6^	0.796	<0.001
**BPD (mm)**			
Dmax (mm)	Y = −0.30 + 6.33*BPD*10^–2^ + 7.14*BPD^3^*10^–7^	0.911	<0.001
Dmin (mm)	Y = 0.187 + 1.545*BPD*10^–2^ + 4.283*BPD2*10^–4^	0.924	<0.001
Diameter distensibility (100%)	Y = 0.8–0.008*BPD + 1.11*BPD^3^*10^–7^	0.799	<0.001
**FL (mm)**			
Dmax (mm)	Y = −0.17 + 8.49*FL*10^–2^ + 1.01*FL^2^*10^–5^	0.917	<0.001
Dmin (mm)	Y = −0.11 + 4.67*FL*10^–2^ + 3.89*FL^2^*10^–4^	0.929	<0.001
Diameter distensibility (100%)	Y = 0.374 + 0.013*FL − 4.39*FL^2^*10^–4^ + 2.99*FL^3^*10^–6^	0.801	<0.001

GA, gestational age; BPD, biparietal diameter; FL, femur length; Dmax, maximum systolic diameter; Dmin, minimum diastolic diameter.

**TABLE 3:** Regression models for the prediction of the SD of Dmax, Dmin, and diameter distensibility based on GA, BPD, and FL.

Parameter	Model derived from regression analysis	*R*	*P*
**GA (weeks)**			
Dmax (mm)	Y = 0.026*GA-0.227	0.357	<0.001
Dmin (mm)	Y = 0.027*GA-0.301	0.383	<0.001
Diameter distensibility (%)	Y = 0.140–0.255*GA*10^–3^	0.259	<0.001
**BPD (mm)**			
Dmax (mm)	Y = 7.761*10^–3^*BPD-0.055	0.291	<0.001
Dmin (mm)	Y = 9.217*BPD*10^–3^-0.203	0.37	<0.001
Diameter distensibility (%)	Y = 0.131–8.040*BPD*10^–3^	0.219	<0.001
**FL (mm)**			
Dmax (mm)	Y = 0.011*FL-0.103	0.345	<0.001
Dmin (mm)	Y = 0.011*FL-0.157	0.398	<0.001
Diameter distensibility (%)	Y = 0.122–9.870*FL*10^–4^	0.249	<0.001

SD, standard deviation; GA, gestational age; BPD, biparietal diameter; FL, femur length; Dmax, maximum systolic diameter; Dmin, minimum diastolic diameter.

Z-scores for each measured value for Dmax, Dmin, and diameter distensibility could readily be calculated as follows: Taking GA and diameter distensibility as examples, Z-score = (observed diameter distensibility– predicted value)/predicted SD (the predicted diameter distensibility and *SD* are available from the equations in Tables 2, 3). An example of a Z-score calculation for a given measurement is as follows: actual measured diameter distensibility = 10.5% at 24 weeks gestation. With the equations in Tables 2, 3, the predicted diameter distensibility at 24 weeks’ GA = −0.98–0.03*GA + 4.48*GA^3*^10^–6^ = 0.344; predicted *SD* of diameter distensibility at 24 weeks’ = 0.14–0.255*GA*10^–3^ = 0.816; and the Z-score = (0.105–0.344)/0.816 = −2.93. Therefore, this fetus had a low diameter distensibility, at 2.93 SD below the predicted mean excursion for a fetus at 24 weeks’ GA, which was out of the normal range.

## Discussion

The elastic properties of the ascending aorta are important components of the arterial system’s elastic nature. These elastic properties play an important role in maintaining normal cardiovascular physiology due to the pulsatile blood flow generated during systole (17, 18). Damage to the elasticity of the ascending aorta will result in a widening of pulse pressure, an increase in mean arterial pressure, and a decrease in coronary blood perfusion (19). Early identification of fetuses with abnormalities in elastic properties is becoming increasingly important.

To the best of our knowledge, this study firstly demonstrated decreased ascending aortic diameter distensibility along with increasing GA in normal fetuses and established the Z-scores models of fetal ascending aortic diameter distensibility.

The present data reveal that aortic diameter distensibility decreases with increasing GA in the normal fetus, indicating decreases in aortic elastic properties with structural maturation during the fetal period. According to zoological research, when fetal blood pressure rises during pregnancy, so does the expression of elastin, and as the elastic fibers gradually transform from a microfibrillar scaffold in early pregnancy to an elastin-microfibril composite, they have increased intrinsic mechanical properties (expressible in material properties such as moduli). To adapt to the strong mechanical load after birth, the aortic wall undergoes mature changes (20).

This result is consistent with previous findings in infants (6) and fetuses (7). Akira and Yoshiyuki (6) analyzed aortic stiffness in both preterm and term infants with appropriate GA and demonstrated that the stiffness index was considerably increased with increasing GA. Taketazu et al. (7) examined phasic variations in ascending aortic diameter along with Doppler flow in normal fetuses with GA between 18 and 38 weeks and found that aortic compliance was dramatically reduced with increasing GA. However, our findings contradicted the report of Miyashita et al. (12). They analyzed abdominal aortic diameter distensibility in 100 normal fetuses and discovered that there was no significant change in aortic diameter distensibility with GA. We speculated the disparity between our study and Miyashita et al.’s report may be related to the discrepancy in the aortic segment and the sample size.

This study employed the Z-score model to reflect the diameter distensibility of the fetal ascending aorta as a multiple of *SD*, and gave the typical 90% range of Z-score, that is, when Z-score is within the ± 1.65 range, the greater the absolute value of Z-score, the greater the deviation from the mean, although it remains within the normal range. If the absolute value of the Z-score is greater than 1.65, the elastic properties of the ascending aorta are thought to be abnormal. In addition to GA, this study incorporated BPD and FL as independent variables to establish the Z-score model of diameter distensibility. It was found that increasing the independent variables more accurately and dynamically reflected the variation in the distensibility index of the ascending aorta with the biological parameters of the fetus itself. For skeletal dysplasia, Z-scores based on GA are the preferred reference due to unreliable measurements of bone structures. For renal malformation or oligohydramnios, Z-scores based on FL are appropriate because they are easy to obtain and relate mostly to fetal size.

Diameter distensibility may possibly be a promising sonographic marker for vascular function in the fetal period since the measurement is straightforward and reproducible. Only a few research have previously established the normal reference value range for the elastic properties of the normal fetal aorta. Moreover, these studies are based on small sample sizes, complex measurement methods, and poor repeatability. Although the potential clinical usefulness of diameter distensibility needs to be further explored, the results presented in this study may serve as a baseline reference for evaluating impaired vascular elastic properties in high-risk pregnancies.

There were several limitations in this study. Firstly, although ECG monitoring in the fetal period cannot define the end-systole and end-diastole timepoints, we confirmed the timepoints as the frame before the aortic valve closes and the frame before the aortic valve opens separately. The high frame frequency (range 180–230 fps) is able to guarantee the accuracy of timepoints. Secondly, despite the fact that the total sample size was sufficient, there were discrepancies among the various GA groups, particularly with fewer cases (<20 cases) collected in less than 20 weeks and larger than 38 weeks.

## Conclusion

In summary, this study revealed that normal fetuses have decreased ascending aortic diameter distensibility with increasing GA and established the Z-scores models of fetal ascending aortic diameter distensibility vs. GA, BPD, and FL. These models have the potential to be beneficial for evaluating aortic elastic properties in the fetal period.

## Data availability statement

The original contributions presented in this study are included in the article/supplementary material, further inquiries can be directed to the corresponding author.

## Ethics statement

The studies involving human participants were reviewed and approved by the Institutional Review Board for Ethics of the Central hospital of Yueyang. The Institutional Review Board for Ethics of the Second Xiangya Hospital of Central South University. Written informed consent to participate in this study was provided by the participants’ legal guardian/next of kin.

## Author contributions

SZ contributed to conception, design of the study, and wrote the first draft of the manuscript. FC, AY, LC, YL, DZ, and SZ organized the database. FC and LY performed the statistical analysis. FC wrote sections of the manuscript. All authors contributed to manuscript revision, read, and approved the submitted version.
